# Supplementary material for: Evaluating the effectiveness of applying aroma seals to masks in reducing stress caused by wearing masks: A randomized controlled trial
Source: PLoS One. 2023 Nov 16;18(11):e0294357. doi: 10.1371/journal.pone.0294357 (PMC10653515; doi:10.1371/journal.pone.0294357)
Supplement: S1 Table — (DOCX) [file pone.0294357.s001.docx]

S1 Table DASS-21 Scores in the Aroma-Seal Use Group and Placebo-Seal Use Group at Baseline, 1 Week, and 2 Week

|  | Aroma-seal use group (n =31) | | | | |  | Placebo-seal use group (n = 30) | | | | |
| --- | --- | --- | --- | --- | --- | --- | --- | --- | --- | --- | --- |
|  | Baseline |  | 1 week |  | 2 week |  | Baseline |  | 1 week |  | 2 week |
| Total Score | 8.00 ± 7.14 |  | 4.10 ± 5.11 |  | 4.42 ± 5.64 |  | 8.67 ± 7.49 |  | 9.27 ± 12.3 |  | 7.50 ± 8.00 |
|  |  |  |  |  |  |  |  |  |  |  |  |
| Depression | 2.94 ± 3.00 |  | 1.19 ± 1.92 |  | 1.68 ± 2.40 |  | 2.60 ± 2.66 |  | 3.03 ± 4.27 |  | 2.50 ± 3.13 |
|  |  |  |  |  |  |  |  |  |  |  |  |
| Anxiety | 1.77 ± 1.89 |  | 1.19 ± 1.49 |  | 0.97 ± 1.47 |  | 2.03 ± 1.99 |  | 2.30 ± 3.96 |  | 1.73 ± 2.23 |
|  |  |  |  |  |  |  |  |  |  |  |  |
| Stress | 3.29 ± 3.51 |  | 1.71 ± 2.66 |  | 1.77 ± 2.55 |  | 4.03 ± 3.80 |  | 3.93 ± 4.78 |  | 3.27 ± 3.52 |
