# Supplementary material for: Evaluating the effectiveness of applying aroma seals to masks in reducing stress caused by wearing masks: A randomized controlled trial
Source: PLoS One. 2023 Nov 16;18(11):e0294357. doi: 10.1371/journal.pone.0294357 (PMC10653515; doi:10.1371/journal.pone.0294357)
Supplement: S2 Table — (DOCX) [file pone.0294357.s002.docx]

S2 Table WHO-5 Scores in Aroma Seal and Placebo Groups at Baseline and 2 Week

|  | Aroma-seal use group (n =31) | | |  | Placebo-seal use group (n = 30) | | |
| --- | --- | --- | --- | --- | --- | --- | --- |
|  | Baseline |  | 2 week |  | Baseline |  | 2 week |
| Total Score | 15.55 ± 4.36 |  | 18.81 ± 4.22 |  | 15.23 ± 3.68 |  | 16.97 ± 3.72 |
|  |  |  |  |  |  |  |  |
| W1 | 3.42 ± 0.81 |  | 3.81 ± 0.79 |  | 3.30 ± 0.79 |  | 3.53 ± 0.97 |
|  |  |  |  |  |  |  |  |
| W2 | 3.23 ± 1.02 |  | 4.03 ± 0.80 |  | 3.23 ± 0.97 |  | 3.60 ± 0.81 |
|  |  |  |  |  |  |  |  |
| W3 | 3.00 ± 1.03 |  | 3.58 ± 1.18 |  | 2.93 ± 1.05 |  | 3.23 ± 0.86 |
|  |  |  |  |  |  |  |  |
| W4 | 2.77 ± 1.15 |  | 3.71 ± 1.30 |  | 2.83 ± 1.29 |  | 3.37 ± 1.16 |
|  |  |  |  |  |  |  |  |
| W5 | 3.13 ± 1.20 |  | 3.68 ± 1.08 |  | 2.93 ± 1.08 |  | 3.23 ± 1.01 |
