# Supplementary material for: Evaluating the effectiveness of applying aroma seals to masks in reducing stress caused by wearing masks: A randomized controlled trial
Source: PLoS One. 2023 Nov 16;18(11):e0294357. doi: 10.1371/journal.pone.0294357 (PMC10653515; doi:10.1371/journal.pone.0294357)
Supplement: S1 File — (DOC) [file pone.0294357.s004.doc]

Clinical trial protocol

| **Clinical trial name** | **:** | Evaluating the effectiveness of applying aroma seals to masks in reducing stress caused by wearing masks: A randomized controlled trial |
| --- | --- | --- |
| **Lead principal investigator** | **:** | Nobuyuki Wakui (Hoshi University) |
| **Interventions** | **:** | Aroma Seal with mask |
| **Research subject** | **:** | University student (18+ years) |
| **Study design** | **:** | Randomized, double-blind, parallel-group comparison trial |
| **Created date** | **:** | Date of creation Jun 29, 2021 |
|  |  | Date of revision July 19, 2021 |
|  |  |  |

# List of abbreviations

| Abbreviation | Full expression |
| --- | --- |
| COVID-19 | Coronavirus Infectious Disease emerged in 2019 |
| CDC | Centers for Disease Control and Prevention |
| WHO-5 | WHO – Five Well-being Index |
| DASS-21 | Depression, Anxiety, and Stress Scale |

**Trial protocol**

1. Significance, purpose, and background of the research

1-1. Please describe the background to establish the research plan, the issues to be solved, and the social significance of the current by referring to previous research

| With the spread of the new coronavirus infection (COVID-19), masks are being worn across the world. The US Centers for Disease Control and Prevention (CDC) recently issued guidelines for people who have completed vaccinations, which state that mask wearing is not necessary for the majority of daily activities but is required during public transportation, including airplanes, airports, and train stations. Moreover, mask wearing will continue to be required in campuses and hospitals [1]. Prior to the spread of COVID-19, the use of masks was limited to the prevention of the spread of influenza and hay fever [2, 3] However, given the post-vaccination action guidelines announced by the CDC, mask wearing is assumed necessary in the foreseeable future [1].  While scholars have demonstrated the effectiveness of masks [4], reports of an increase in the number of people who feel stressed by wearing masks have recently emerged. In fact, more than 80% of people have experienced a few forms of stress such as difficulty in breathing, rough skin, or heat [5]. Therefore, countermeasures against stress due to mask wearing are also important for improving quality of life.  Essential oils can be used as a countermeasure for stress due to mask wearing [6–8]. Popular as a non-invasive and natural approach [9], essential oils are known to produce a calming effect and are considered effective in relieving stress [10–15]. Spraying essentials or attaching aroma stickers on masks can be a method for relieving stress. In fact, the results of research on spraying essential oils on masks have demonstrated that doing so improves the quality of sleep in nurses after night shifts [16] and reduces anxiety in patients undergoing dialysis [17].  Alternatively, the use of Aroma Seal is expected to exert the same effect as aroma spray in relieving stress. In particular, in contrast to spraying, stickers are expected to produce a lasting effect, because essential oils are less likely to volatilize. However, this tendency lacks scientific verification, such that the extent to which Aroma Seal can relieve stress due to mask wearing remains unclear.  Therefore, this study designed a double-blind randomized controlled trial to elucidate the stress-relieving effect of Aroma Seal. The researchers hope that this research will illustrate the usefulness of Aroma Seal and contribute to the improvement of quality of life. |
| --- |

1-2. Please describe a corresponding list of prior research and literature that you commented on, cited, or referenced in “1-1.”

| 【1】CDC, <https://www.cdc.gov/coronavirus/2019-ncov/vaccines/fully-vaccinated.html>  Accessed: 2021.06.15  【2】Qualls N, Levitt A, Kanade N, Wright-Jegede N, Dopson S, Biggerstaff M, Reed C, Amra Uzicanin, Community Mitigation Guidelines to Prevent Pandemic Influenza - United States, MMWR Recomm Rep., 66, 1-32, 2017  【3】Sözener Z, Öztürk B, Aydın Ö, Demirel Y, Pınar N, Bavbek S, Sin B, Mungan D, Coincidence of pollen season and coronavirus disease 2019 pandemic: less time outdoors – lesser allergy symptoms in 2020, Asia Pac Allergy.,11, e16, 2021  【4】Rader B, White L, Burns M, Chen J, Brilliant J, Cohen J, Shaman J, Brilliant L, Kraemer M, Hawkins J, Scarpino S, Astley C, Brownstein J, Mask-wearing and control of SARS-CoV-2 transmission in the USA: a cross-sectional study, Lancet Digit Health., 3, e148-e157,2021.  【5】I sympathize with the “mask stress” that I just heard again! Things that feel plain and painful in a prolonged mask life,　<https://news.yahoo.co.jp/articles/8a9f3be828a321f0a90a7d3357c2069d548d40a1>  Accessed: 2021.06.15  【6】Ahmad R, Naqvi A, Al-Bukhaytan H, Al-Nasser A, Al-Ebrahim A, Evaluation of aromatherapy with lavender oil on academic stress: A randomized placebo controlled clinical trial, Contemp Clin Trials Commun., 14,100346, 2019.  【7】Howard S, Hughes B, Expectancies, not aroma, explain impact of lavender aromatherapy on psychophysiological indices of relaxation in young healthy women, Br J Health Psychol., 13, 603-17,2008.  【8】[The effects of aromatherapy on stress and stress responses in adolescents], J Korean Acad Nurs., 39, 357-65, 2009.  【9】Chen C, Fang H, Fang L, The effects of aromatherapy in relieving symptoms related to job stress among nurses., International Journal of Nursing Practice, 21, 1, 87-93, 2015.  【10】Itai T., Amayasu H., Kuribayashi M., Kawamura N., Okada M., Momose A., Tateyama T., Narumi K., Uematsu W., Kaneko S. Psychological effects of aromatherapy on chronic hemodialysis patients., Psychiatr. Clin. Neurosci., 54, 393-397, 2000.  【11】Sung N, Eun Y, The effect of aromatherapy on stress of nurses working in operating room., J. Kor. Acad. Adult. Nurs., 19, 1-11, 2007.  【12】Seo J, The effects of aromatherapy on stress and stress responses in adolescents., J. Kor. Acad. Nurs., 39, 357-365, 2009.  【13】Chang M, Shen W, Aromatherapy benefits autonomic nervous system regulation for elementary school faculty in Taiwan., Evid. Based Complement Altern. Med., 7 pages, 2011.  【14】Han H, Hur H, Kang Y, Effects of aromatherapy on the stress response of college women with dysmenorrhea during menstruation., J. Kor. Acad. Nurs., 32, 317-326, 2002.  【15】Ali B, Wabel N, Shams S, Ahamad A, Khan S, Anwar F, Essential oils used in aromatherapy: a systemic review, Asian Pacific Journal of Tropical Biomedicine., 5, 601–611, 2015.  【16】Nasiri A, Boroomand M, The effect of rosemary essential oil inhalation on sleepiness and alertness of shift-working nurses: A randomized, controlled field trial, Complementary Therapies in Clinical Practice, 43, 101326, 2021.  【17】Sung S.N., Eun Y. The effect of aromatherapy on stress of nurses working in operating room. J. Kor. Acad. Adult. Nurs.,19, 1–11, 2007. |
| --- |

1-3. Please describe the preliminary research and preparations for this research before making this research plan.

| After conducting a pilot test, we determined that the following evaluation scales are appropriate.  [Stress evaluation]  ・WHO-5 (mental health status table)  → Assesses mental health.  ・DASS-21 (Depression, Anxiety and Stress Scale)  → Evaluates depression, anxiety, and stress. |
| --- |

2. Hypotheses, endpoints and criteria, predictors and outcomes to be demonstrated by this study

*2-1. Please describe the specific goals to be achieved in the research subject to this ethical review.*

| We aim to elucidate whether or not stress due to mask wearing can be alleviated using Aroma Seal.  ・The number of participants is estimated to be 60 undergraduate students.  ・Appropriate tests will be conducted for each item in the questionnaire, and the obtained results will be examined.  →Rating scales for stress:  (1) WHO-5 (mental health condition list)^*1^  (2) DASS-21 (Depression, Anxiety, and Stress Scale)^*2^  We will conduct a randomized, double-blind, parallel-group comparison study to examine the efficacy of Aroma Seal in healthy adults using the abovementioned evaluation scales (1) and (2). We will compare the mental health status of the group using Aroma Seal and another group using placebo to confirm its effectiveness. In addition, we will ensure safety by comparing the incidence of adverse events.  *1. WHO—Five Well-being Index, WHO Collaborating Center in Mental Health.  → WHO-Five is a rating scale that assesses mental health status two weeks prior and is widely used worldwide.  *2. Antony, M.M., Bieling, P.J., Cox, B.J., Enns, M.W., Swinson, R.P., Psychometric properties of the 42-item and 21-item versions of the Depression Anxiety Stress Scales in clinical groups and a community sample. Psychol. Assess. 10, 176–181, 1998.  → DASS-21 has been depicted to be useful as an evaluation scale for depression, anxiety, and stress and is used all over the world. |
| --- |

2-2. Please describe in an easy-to-understand manner the items that can be evaluated objectively when verifying the hypothesis, or the composite index of the items and the evaluation criteria, together with the theoretical basis.

| The independent and dependent variables are as follows.  ***Independent variables***  Gender, age, and difficulty in breathing during mask wearing; we used lifestyle habits as adjustment variables.  ***Dependent variables***  1) WHO-5 (mental health status table)  Items are rated using a six-point scale. Scores range from 0 to 5, and the total score is the sum of all scores. Analysis will be conducted to assess changes from baseline using a mixed-effect model.  2) DASS-21 (Antidepressant, Anxiety, and Stress Rating Scale)  Items are rated using a four-point scale. Scores range from 0 to 3, and the total score is the sum of all scores. Analysis will be conducted to assess change from baseline using the mixed-effect model.    3) Shortness of breath during mask wearing  Items are rated from 1 to 5 points using five methods. Analysis will be focused on assessing change from baseline using the mixed-effect model.  Based on the values obtained from the abovementioned evaluation scales 1, 2, and 3, the study will be conducted a comparison between the experimental and control groups. Specifically, the baseline value is subtracted from the value at each evaluation time point, and the obtained value is used to compare the difference in the degree of change from the baseline value between groups. The independent variables are used to adjust for confounding factors. |
| --- |

3. Safety of this study procedure

| Although the possibility is extremely low, rough skin may occur due to irritation. If symptoms occur, we will recommend medical attention. |
| --- |

4. Subjects and research method

Procedures for conducting the research and details of samples to be collected or data to be collected

4-1. Please specifically describe the attributes, gender, age group, and number of research subjects.

| Attribute: university students  Sex: Man, Woman  Age: 18+ years  Number of people: 60 |
| --- |

4-2. Please write down the overall picture of the subject, the control group, and the breakdown of each experimental group in an easy-to-understand manner.

| This study will designate an experimental (A) and a control (B) group for the intervention study.  Overall picture of the subject: a randomized, double-blind, parallel-group comparison study will be conducted by dividing the participants into two groups, namely, A and B.  Group A (30 cases): group using Aroma Seal  Group B (30 cases): group using placebo seal  ・Inclusion criteria  Those who received a sufficient explanation about the purpose and contents of this research  possess the ability to consent, voluntarily participate, and agree to participate in writing.  ・Exclusion criteria  (1) Those who refused to participate in the research.  (2) Those who use aroma in their daily lives.  (3) Those who are not good at smelling aroma.  (4) Those who are allergic to aroma.  (5) Those with chronic diseases (e.g., hypertension and epilepsy.  6) Those with a mental illness that requires drug therapy.  7) Those who exhibit an allergic reaction during the patch test at the time of test registration.  8) Those who do not feel any stress due to mask wearing at the start of the survey.  9) Those who are considered ineligible by the principal investigator.  ・Usage  　 Aroma Seal　　　1 seal once per day; usage time: from morning until getting home  Use when stressed  　　　　　　　 [Aroma seal for masks: orange–lime scent]  　 Placebo seal　　 1 seal once per day; usage time: from morning until getting home  　　　　　　　　　　　　　　　　　　　　　　　 　 Use when stressed  　　　　　　 　[Seal only: Round seal for aroma seal]  ・Test period  The test period will last 4 weeks in total. Screening will occur across 2 weeks followed by 2 weeks  of continuous use as treatment periods. The schedule for the usage period is as follows. |
| --- |

4-3. Please specifically describe the research implementation procedure and the burden on the subject.

| (1) Implementation procedure  The survey will be conducted after approval by the research ethics committee in accordance with the research ethics regulations of Hoshi Pharmaceutical University.  The participants will be 60 undergraduate students of this university who are aged 18 years or older. They will be divided into two groups, namely, A and B, and a randomized, double-blind, parallel-group comparison study will be conducted. Groups A and B will be screened for 2 weeks, after which group A will continue to use Aroma Seal, and group B will continue to use the placebo seal for 2 weeks. The frequency of use is one sticker per day, and usage time is from morning until returning home. The total study period will be 4 weeks. The intervention period will be 2 weeks. After confirming that the person in charge of monitoring is in an anonymized state, the results of the survey will be aggregated and analyzed at Hoshi Pharmaceutical University. The survey schedule is described in the following figure. At the time of obtaining consent, the participants will fill out the questionnaire to confirm variations in lifestyle habits and health conditions. In addition, a patch test will be conducted to confirm the presence or absence of allergies associated with the test. Only those without an allergic reaction in the patch test will be enrolled in the study. Participants will be randomly assigned to one of the two groups (intervention or control) in a 1:1 ratio. The survey will be conducted in a double-blind manner so that participants and the interventionists, who will be also the data analysts of the present study, cannot discriminate between the groups of participants. Randomization will be perform using the permuted block method with a block size of 2 and 4. The blinding will be maintained until the statistical analysis of the resulting data will be completed.    (2) Concerning the burden of the target person  (2-1) Burden on research subjects and expected risks (including possible adverse events)  ・Burden is associated with filling out questionnaires and participating in research.  ・A possibility exists that rough skin may occur due to the incorrect application of the sticker.  (2-2) Comprehensive evaluation and measures for minimizing burden and risk  ・To minimize burden, a check-type questionnaire is used.  ・To conduct the test safely and appropriately, we will prepare an instruction manual that  summarizes the method of use and explains and distribute information to all participants.  ・By conducting a patch test at the time of research registration, only those without an  allergic reaction to aroma will be included in the test. |
| --- |

4-4. Describe specifically the location, method and amount of sample collection, and what to test from the collected sample.

| We will not collect samples. |
| --- |

5. Basis for Scientific Rationality of Research

(1) Reason for using aroma seal

Using Aroma Seal is expected to relieve stress, and various products have been put on the market in recent years. However, Aroma Seal lacks scientific verification, such that the extent to which it relieves stress due to mask wearing remains unclear.

(2) Research design

1) Reasons for selecting a parallel-group comparison study

・Previous research on aromatherapy and mask wearing have adopted a parallel-group comparison study and demonstrated its usefulness.

・The causal relationship can be elucidated by comparing two groups, namely, the intervention and control groups, at the same time.

2) Reason for the intervention method (intervention between morning and returning home)

In addition to the relaxing effect of aroma, scholars propose that orange/lime produces a refreshing effect, such that using Aroma Seal of essential oils that contain orange/lime when going out will improve the suffocation felt when wearing a mask. As a result, stress relief can be expected.

(3) Reason for the selection of evaluation scale

Rating Scale for Stress:

・WHO-5 (mental health status table)

・DASS-21 (Depression, Anxiety and Stress Scale)

To date, many studies on stress have used the abovementioned evaluation scale. In addition, given the test period, using the abovementioned evaluation scale, which can evaluate mental health conditions within a short period of time, is appropriate.

6. Potential harm in research

| Skin trouble may occur if the aroma or adhesive of the seal adheres to the skin. |
| --- |

7. Benefit to society and Benefits received by the subject through participation

| (1) Benefits to society  By attaching an aroma sticker to masks, the study expect that mental stress due to mask wearing will be alleviated. In addition, by widely reporting the results to society, the study expects that the mental stress of more people will be alleviated.  (2) Benefits received by the target  Using Aroma Seal on masks, people can relax physically and mentally and improve their mood. In addition, alleviating suffocation due to mask wearing is possible when attaching the Aroma Seal. |
| --- |

8. How to deal with harm to research subjects

| (1) Response to subjects when adverse events occur  Sufficient monitoring of the safety of subjects will be instituted during the study.  We will inform the participants to discontinue the use of the seal if harm occurs. In addition, if symptoms persist, we will recommend consultation in a medical institution. The subject is informed if the intervention is discontinued or treatment for adverse events becomes necessary. If it is determined to be an adverse occurrence, we will bear the full cost of therapy.  (2) Other adverse events  Other adverse events will be appropriately documented by the investigator in the record book. |
| --- |

9. Study change, discontinuation/suspension

| (1) Change of the study  Any changes or alterations to the research implementation protocol or informed consent document for this study must be first approved by the Ethics Committee.  (2) Discontinuation or suspension of the study  The researcher will consider whether to continue the study if any of the following applies.  1) When it is judged that it is extremely difficult to enroll the subjects and reach the planned  goal in several cases.  2) When the Ethics Committee instructs to change the implementation plan and it is  determined that it is difficult to accept this.  If the Ethics Committee recommends or instructs the lead investigator to cease the research, the principal investigator must do so. |
| --- |

10. Please indicate the research implementation period.

| The research implementation period is planned from after approval by the ethics communitee to March 31, 2024. |
| --- |

11. Please describe the statistical analysis.

| In this test, a comparison between the groups will be made between the Aroma and placebo seals.  Descriptive statistics will be used to present the demographic characteristics of the participants. Means and standard deviations will be used to provide numerical data, whereas frequencies and ratios will be used to provide categorical data.  To determine the improvement in the primary endpoint of the scale for stress, changes in DASS-21 scores measured at the time points from the scores measured at baseline plan to assess in each group by using a mixed-effects model of analysis of covariance with baseline values as covariates.  To assess the enhancement in mental health through stress reduction caused by wearing a mask, changes in WHO-5 scores in each use group at the time of measurement (week 2) concerning baseline values will be evaluated using a mixed-effects model of analysis of covariance with baseline values as covariates.  To see if wearing a mask eased suffocation, the user groups will be evaluated for improvement in the mask-suffocation score using a mixed-effects model of the analysis of covariance with baseline values as covariates  For each usage group, the number of participants who encountered adverse events and the incidence of adverse events will be documented for safety evaluation.  A p-value of less than 0.05 for all tests will be considered statistically significant. All analyses will be conducted using a two-tailed test. A two-sided 95% confidence interval is used. |
| --- |

12. Please describe the consideration of the human rights of subjects and the method of protection of personal information.

| When conducting this research, it will be started after being reviewed by the Research Ethics Committee of Hoshi Pharmaceutical University. We shall conduct this research following the Helsinki Declaration (updated in October 2013, Fortaleza), the Ethical Guidelines for Life Science and Medical Research Involving Human Subjects (revised on March 10, 2022), and other applicable laws and regulations. The applicant must provide the subject with a research consent form approved by the Hoshi University Research Ethics Committee, provide an adequate explanation in writing and orally, and take ethical considerations into account, such as obtaining the subject's written consent to participate in the research of his/her own free will.  When handling informationrelated to research implementation, a number unrelated to the subject's personal information will be allocated and handled with due regard for the subject's confidentiality. Also, when publishing research results, exclude information that could identify the subjects. We do not use the samples collected from the subjects for any purpose other than the research. |
| --- |

13. Please describe the method for obtaining consent.

| The researcher handed over the informed consent document approved by the review committee to the subject,  Provide sufficient oral explanations and obtain the subject's voluntary consent in writing. |
| --- |

14. Please describe the cost burden of the subject.

| Since this research is covered by the research expenses of the Hoshi Pharmaceutical University, there is no cost burden on the subjects. |
| --- |

15. Please describe the record keeping and publication of research results.

| The primary investigator should guarantee that critical documents linked to research execution (copy of application documents, a notification from the ethics committee, copy of various application forms and reports, subject identification code list, consent form, other documents, records necessary to guarantee data reliability) are kept until 5 years after the discontinuation or completion of the research, after which personal information will be carefully discarded. When publishing the findings of this study by presenting them at associated academic societies, we shall take care to preserve personal information so that the subjects are not identified. |
| --- |

16. Please describe the cost burden of the subject.

| Since this research is covered by the research expenses of the Hoshi Pharmaceutical University, there is no cost burden on the subjects. |
| --- |

17. Please describe research funding and conflicts of interest.

| This research did not receive any specific grant from funding agencies in the public, commercial, or  not-for-profit sectors. |
| --- |

18. Please describe the research implementation system.

| (1) Principal investigator (plays a central role in the operation and management of the study by preparing draft protocols (study protocols), questionnaires, consent documents, etc.)  ・Nobuyuki Wakui (Hoshi University)  ・Kotoha Ichikawa (Hoshi University)    (2) Research Data Management Supervisor (responsible for the operation of the study together with the Principal Investigator and supervises the study to ensure that it runs smoothly, impartially, and neutrally.)  ・Miho Yamamura (Hoshi University)  (3) Research collaborator  ・Aika Okami (Hoshi University)  ・Hinako Kagi（Hoshi University）  ・Shoko Kawakugo（Hoshi University）  ・Chikako Togawa (Hoshi University)  ・Raini Matsuoka (Hoshi University)  ・Mai Watanabe (Hoshi University)  ・Shunsuke Shirozu (Hoshi University)  ・Yuika Tsubota (Hoshi University)  ・Yukiko Yoshizawa (Hoshi University)  ・Yoshiaki Machida（Hoshi University）  (4) Study statistical analysis  ・Nobuyuki Wakui (Hoshi University)  ・Kotoha Ichikawa (Hoshi University) |
| --- |

19. Please describe your contact information.

| **Inquiries regarding research plans**  　　Lead principal investigator:  Name: Nobuyuki Wakui  Affiliation: Division of Applied Pharmaceutical Education and Research, Faculty of Pharmaceutical Sciences  Hoshi University, 2-4-41 Ebara, Shinagawa-ku, Tokyo 142-8501, Japan  Phone No: 03-5498-5760  Fax No: 03-5498-5760  Email Address: n-wakui@hoshi.ac.jp |
| --- |
