# Supplementary material for: Evaluating the effectiveness of applying aroma seals to masks in reducing stress caused by wearing masks: A randomized controlled trial
Source: PLoS One. 2023 Nov 16;18(11):e0294357. doi: 10.1371/journal.pone.0294357 (PMC10653515; doi:10.1371/journal.pone.0294357)
Supplement: S2 File — (DOC) [file pone.0294357.s005.doc]

臨床試験実施計画書

| **臨床試験名** | **:** | マスク着用時のストレス緩和を目的としたアロマシール利用による有効性の評価：並行群間比較試験 |
| --- | --- | --- |
| **研究責任者** | **:** | 湧井 宣行（星薬科大学） |
| **介入内容** | **:** | マスクへのアロマシールの貼付 |
| **対象者** | **:** | 18歳以上の大学生 |
| **試験デザイン** | **:** | 二重盲検、無作為化並行群間比較試験 |
| **作成日** | **:** | 2021年6月29日 作成 |
|  |  | 2021年7月19日 改訂 |

# 略語リスト

| 略語 |  |
| --- | --- |
| COVID-19 | 新型コロナウイルス感染症 |
| CDC | アメリカ疾病対策センター |
| WHO-5 | WHO-5 精神的健康状態表 |
| DASS-21 | 抑うつ、不安、ストレス評価尺度 |

**試験プロトコル**

1.この研究の意義・目的・背景など

1-1.この研究計画を立てた背景事情と、この研究によって解決しようとする課題とそれによる社会的意義について、先行研究・文献を参考にしながら述べてください。

| 新型コロナウイルス感染症（COVID-19）の蔓延にともない、世界中でマスクが着用されている。アメリカ疾病対策センター（CDC）が最近発表したワクチン接種を完了した人向けの行動指針では、日常生活の大半でマスクの着用は不要であるとしたものの、航空機を含む公共交通機関、空港・駅の構内、病院などでは今後もマスクの着用を必要としている【1】。COVID-19が蔓延する前は、インフルエンザの感染予防や花粉症対策などのみにマスクの利用は限られてきた【2, 3】。しかし、CDCが発表したワクチン接種後の行動指針示を考慮すると、今後もしばらくの間はマスクの着用が必要であると考えられる【1】。  マスクによる有効性が示されている【4】一方で、昨今ではマスクの着用によってストレスを感じている人の増加も報告されている。実際、8割以上の人がマスクの着用によって、息苦しい、肌が荒れる、暑いなどの何らかのストレスを感じた経験があるされている【5】。よって、マスク着用により生じるストレスへの対策も多くの人の生活の質を高める上で重要である。  マスク着用によるストレス対策としては、アロマ精油の利用がある【6-8】。非侵襲的で自然なアプローチとして親しまれているアロマ精油は【9】、心を落ち着かせる効果などが知られており、日常生活でのストレス緩和に有効であるとされている【10-15】。マスク着用によるストレスの緩和方法としては、マスクへのアロマスプレーの噴霧やアロマシールの貼付がある。実際、マスクへアロマスプレーを噴霧した研究結果では、夜勤後の看護師の睡眠の質を高めたり【16】、透析患者の不安を軽減させたりしている【17】。  一方、アロマシールの利用については、アロマスプレーと同様にストレスを緩和させる効果が期待されている。特に、スプレーの噴霧とは異なり、シールではアロマ精油が揮発しにくいため持続効果が期待できる。しかし、未だ科学的な検証は行われておらず、実際、どの程度マスク着用によるストレスをアロマシールが緩和させるかは明らかにされていない。  そこで本研究では、アロマシールの利用によるマスク着用時のストレス緩和効果を明らかにするため、二重盲検無作為化比較試験（RCT）を計画した。本研究の実施によりアロマシールの有用性が示され、多くの人の生活の質向上に貢献することを期待している。 |
| --- |

1-2.「1-1」にて解説・引用または参照した先行研究・文献について、対応するリストを作成してください。

| 【1】CDC, <https://www.cdc.gov/coronavirus/2019-ncov/vaccines/fully-vaccinated.html>  Accessed: 2021.06.15  【2】Qualls N, Levitt A, Kanade N, Wright-Jegede N, Dopson S, Biggerstaff M, Reed C, Amra Uzicanin, Community Mitigation Guidelines to Prevent Pandemic Influenza - United States, MMWR Recomm Rep., 66, 1-32, 2017  【3】Sözener Z, Öztürk B, Aydın Ö, Demirel Y, Pınar N, Bavbek S, Sin B, Mungan D, Coincidence of pollen season and coronavirus disease 2019 pandemic: less time outdoors – lesser allergy symptoms in 2020, Asia Pac Allergy.,11, e16, 2021  【4】Rader B, White L, Burns M, Chen J, Brilliant J, Cohen J, Shaman J, Brilliant L, Kraemer M, Hawkins J, Scarpino S, Astley C, Brownstein J, Mask-wearing and control of SARS-CoV-2 transmission in the USA: a cross-sectional study, Lancet Digit Health., 3, e148-e157,2021.  【5】I sympathize with the “mask stress” that I just heard again! Things that feel plain and painful in a prolonged mask life,　<https://news.yahoo.co.jp/articles/8a9f3be828a321f0a90a7d3357c2069d548d40a1>  Accessed: 2021.06.15  【6】Ahmad R, Naqvi A, Al-Bukhaytan H, Al-Nasser A, Al-Ebrahim A, Evaluation of aromatherapy with lavender oil on academic stress: A randomized placebo controlled clinical trial, Contemp Clin Trials Commun., 14,100346, 2019.  【7】Howard S, Hughes B, Expectancies, not aroma, explain impact of lavender aromatherapy on psychophysiological indices of relaxation in young healthy women, Br J Health Psychol., 13, 603-17,2008.  【8】[The effects of aromatherapy on stress and stress responses in adolescents], J Korean Acad Nurs., 39, 357-65, 2009.  【9】Chen C, Fang H, Fang L, The effects of aromatherapy in relieving symptoms related to job stress among nurses., International Journal of Nursing Practice, 21, 1, 87-93, 2015.  【10】Itai T., Amayasu H., Kuribayashi M., Kawamura N., Okada M., Momose A., Tateyama T., Narumi K., Uematsu W., Kaneko S. Psychological effects of aromatherapy on chronic hemodialysis patients., Psychiatr. Clin. Neurosci., 54, 393-397, 2000.  【11】Sung N, Eun Y, The effect of aromatherapy on stress of nurses working in operating room., J. Kor. Acad. Adult. Nurs., 19, 1-11, 2007.  【12】Seo J, The effects of aromatherapy on stress and stress responses in adolescents., J. Kor. Acad. Nurs., 39, 357-365, 2009.  【13】Chang M, Shen W, Aromatherapy benefits autonomic nervous system regulation for elementary school faculty in Taiwan., Evid. Based Complement Altern. Med., 7 pages, 2011.  【14】Han H, Hur H, Kang Y, Effects of aromatherapy on the stress response of college women with dysmenorrhea during menstruation., J. Kor. Acad. Nurs., 32, 317-326, 2002.  【15】Ali B, Wabel N, Shams S, Ahamad A, Khan S, Anwar F, Essential oils used in aromatherapy: a systemic review, Asian Pacific Journal of Tropical Biomedicine., 5, 601–611, 2015.  【16】Nasiri A, Boroomand M, The effect of rosemary essential oil inhalation on sleepiness and alertness of shift-working nurses: A randomized, controlled field trial, Complementary Therapies in Clinical Practice, 43, 101326, 2021.  【17】Sung S.N., Eun Y. The effect of aromatherapy on stress of nurses working in operating room. J. Kor. Acad. Adult. Nurs.,19, 1–11, 2007. |
| --- |

1-3.この研究計画を立てるまでの予備的研究およびこの研究の準備状況について記してください。

| 本研究に関するパイロット試験を行った結果、以下の評価尺度が妥当であると判断した。  【ストレス評価】  ・WHO-5（精神的健康状態表）  　→精神健康状態を評価する。  ・DASS-21（うつ、不安、ストレス評価尺度）  　→抑うつ、不安、ストレスを評価する。 |
| --- |

2.この研究によって実証しようとする仮説･エンドポイントと判定基準、予測因子とアウトカム

*2-1.今回の倫理審査の対象となる研究において達成すべき具体的な目標を述べてください。*

| 本研究ではアロマシールの利用によりマスク着用時のストレスが緩和するか明らかにする。  ・参加者数は、本学学部生60名を予定している。  ・アンケートの各項目に対して適切な検定を行い、得られた結果について検討する。  →ストレスに関する評価尺度：  ①　WHO-5（精神的健康状態表）※1  ②　DASS-21（うつ、不安、ストレス評価尺度）※2  ③ マスク着用による息苦しさ（4段階のリッカート尺度）  上記、①、②、③の評価尺度を用いて健康成人に対し、アロマシールの有効性を検討するため、無作為化、二重盲検、並行群間比較試験を実施する。アロマシール使用群とプラセボ使用群の精神的健康状態を比較し、有効性を確認する。また、有害事象の発現割合を比較して安全性を確認する。  ※1. WHO-5 精神的健康状態表、WHO Collaborating Centre in Mental Health.  → 2週間前の精神的健康状態を評価する評価尺度であり、世界中で使用されている。  ※2. Antony, M.M., Bieling, P.J., Cox, B.J., Enns, M.W., Swinson, R.P., Psychometric  properties of the 42-item and 21-item versions of the Depression Anxiety Stress Scales  in clinical groups and a community sample. Psychol. Assess. 10, 176–181, 1998.  → DASS-21は抑うつ、不安、ストレスの評価尺度として有用性が示されており、世界中で使用されている。 |
| --- |

2-2.仮説を検証する際に客観的に評価できる項目または項目の合成指標とその判定基準について、理論的根拠とあわせてわかりやすく述べてください。

| 評価判定時に用いる独立変数・従属変数は、以下の通り設定する。  独立変数  性別、年齢、マスクの息苦しさ、マスク使用時間、生活習慣等に関する情報を調整変数とする。  従属変数  1）WHO-5（精神的健康状態表）  回答は5件法で、0点から5点として集計し、Total Scoreは全ての点数を合算する。解析は混合効果モ  デルを使用してベースラインからの変化を評価する。  2）DASS-21（抗うつ、不安、ストレス評価尺度）  回答は4件法で、0点から3点として集計し、Total Scoreは全ての点数を合算する。解析は混合効果モ  デルを使用してベースラインからの変化を評価する。  3）マスク着用による息苦しさ  回答は 4件法で 1点から 4点で評価する。解析は混合効果モデルを使用してベースラインからの変化  を評価する。  上記 1、2、3の評価尺度から得られた値をもとに、実験群と対照群とで群間比較を行う。  具体的には、各評価時点の値からベースライン値を引き、得られた値を用いて群間でベースライン値からの変化量の差を比較する。なお、独立変数は交絡因子の調整に用いる。 |
| --- |

3.この研究の手続の安全性について記載してください。

| 本研究では、極めて可能性としては低いが、皮膚刺激による肌荒れを起こす可能性がある。症状が発生した場合は、医療機関の受診を勧める。 |
| --- |

4.対象者および研究方法について

研究の実施手順（研究分担および場所も含む）と採取する試料または収集するデータの詳細

4-1.研究対象者の属性、性別、年齢層、人数について具体的に記載してください。

| 属性：本学学部生  性別：男・女  年齢層：18 歳以上  人数： 60名 |
| --- |

4-2.対象者の全体像、対照群、実験群ごとの内訳等わかりやすく記載してください。

| 本研究では、介入試験のため、実験群（A）・対照群（B）を設定する。  対象者の全体像：試験参加者をA群とB群の2群に分け、無作為化、二重盲検、並行群間比較試験を  実施する。  A群（30例）：アロマシール使用群  B群（30例）：プラセボ使用群  ・選択基準  本研究の参加にあたり十分な説明を受けた後、十分な理解の上、本人の自由意志による文書同意が得  られた方  ・除外基準  (1) 本人の意思により研究への参加を拒否した方  (2) アロマを普段の生活で使用している方  (3) アロマの匂いが苦手な方  (4) アロマにアレルギーのある方  (5) 高血圧やてんかんなどの慢性疾患をもっている方  (6) 薬物療法を必要とする精神疾患を合併している方  (7) 試験登録時のパッチテストでアレルギー反応が認められた方  (8) 調査開始時点でマスク着用によるストレスを全く感じていない方  (9) 臨床試験実施者が研究への組み入れを不適切と判断した方    ・使用方法  　 アロマシール　　1日1回　1回1シール　使用時間：朝から帰宅するまで  　　　　　　　【マスク用アロマシール：　オレンジ／ライムの香り】  　 プラセボ　　　　1日1回　1回1シール　使用時間　朝から帰宅するまで  　　　　　　　【シールのみ：アロマシール用ラウンドシール】  ・試験期間  試験期間は全4週間とする。2週目までをスクリーニング週とし、その後、2週間の継続使用週を投  与期間とする。なお、使用期間のスケジュールは以下の通りである。 |
| --- |

4-3. 研究の実施手順、対象者の負担について具体的に記載してください。

| (1) 実施手順  　調査は、星薬科大学（以下、本学）の研究倫理規定に基づき、研究倫理委員会の承認後に実施する。  　調査対象は、18歳以上の本学学部生60名であり、実施にあたって、対象者をA群とB群の2群に分け、無作為化、二重盲検、並行群間比較試験を実施する。A・B群は2週間のスクリーニングを行った後、A群にアロマシール、B群にプラセボシールを2週間継続使用する。使用回数は1日1回1シールの使用とし、使用時間は朝から帰宅時までとする。試験期間は全4週間とする。このうち、介入期間は2週間である。取得した調査票の結果は、モニタリング担当者が匿名化状態であるのを確認後、星薬科大学にてデータの集計・解析を行う。以下に、調査スケジュールを記載する。なお、同意取得時には研究対象者個人間の生活習慣等のばらつきや健康状態の確認のため、問診票の記入を行う。また、試験実施に伴うアレルギーの有無確認のため、パッチテストを実施する。本試験には、パッチテストでアレルギー反応のない方のみを試験登録する。    (2) 対象者の負担について  （2-1）研究対象者に生じる負担並びに予測されるリスク（起こり得る有害事象を含む）  ・調査票の記入や研究参加に伴う負担が強いられる。  ・シールの貼り間違えなどによる肌荒れが起こる可能性がある。  （2-2）総合的評価並びに当該負担及びリスクを最小化する対策  　・負担を最小限にするため、チェック式の調査票を用いている。  　・安全かつ適切な試験実施のため、使用方法についてまとめた説明書を作成し、試験参加者全員に  説明および配布する。  ・研究登録時にパッチテストを実施することで、アロマに対してアレルギー反応がない方のみ  試験対象者とする。 |
| --- |

4-4. 試料を採取する場所や方法、量、そして採取した試料から何を検査するのかなどを具体的に記載してください。

| 試料の採取は行わない。 |
| --- |

5.研究の科学的合理性の根拠

1）アロマシール使用の理由

アロマシールの利用については、ストレスを緩和させる効果が期待され、近年、様々な商品が発売されている。しかし、未だ科学的な検証は行われておらず、実際、どの程度マスク着用のストレスをアロマシールが緩和させるか明らかにされていない。

2）研究デザインについて

(1) 並行群間比較試験の選択理由

・これまでのアロマテラピーとマスクに関する研究で、並行群間比較試験を採用しており、有用性が示されているため。

・同時期に介入群と対照群の2群に分けて比較を行うことで、因果関係を明らかにすることができる。

(2) 介入方法（朝から帰宅までの時間に介入）の理由

アロマのリラックス効果に加え、オレンジ／ライムのリフレッシュ効果があるとされていることから、オレンジ／ライム含有の精油のアロマシールを外出時に使用することでマスク着用時の息苦しさを改善させ、ストレス緩和が期待できるため。

3）評価尺度選択の理由

ストレスに関する評価尺度：

・WHO-5（精神的健康状態表）

・DASS-21（うつ、不安、ストレス評価尺度）

これまでのストレスに関する研究の多くは、上記評価尺度が用いられている。また、本研究での試験期間を考慮すると、短期間で精神的健康状態を評価できる上記評価尺度の利用が妥当である。

6.研究に伴う危害の可能性

| シールのアロマや粘着剤が肌に付着した場合、皮膚トラブルが生じる可能性がある。 |
| --- |

7.社会への便益と参加により対象者の受ける利益

| (1) 社会への便益  マスクにアロマシールを貼付することで、マスク着用による精神的ストレスの緩和が見込まれる。また、その結果を社会に広く報告することで、より多くの人の精神的ストレスを緩和することが見込まれる。  (2) 対象者の受ける利益  　マスクにアロマシールを使用することで、心身ともにリラックスし、気分が改善することができる。また、アロマシールの貼付時にマスクによる息苦しさを改善することができる。 |
| --- |

8. 研究対象者に危害が起きた時の対処方法

| (1) 有害事象発生時の被験者への対応  研究中は研究対象者の安全性について十分なモニタリングを行う。有害事象が発生した場合にはシールの使用を中止するよう参加者に通知する。 なお、症状が続く場合には医療機関への受診を進める。 介入が中止される場合、または有害事象の治療が必要になる場合には対象者に通知する。  (2) その他の有害事象  研究者は全ての有害事象を記録簿に適切に記録する。 |
| --- |

9. 研究の変更、中止について記載してください。

| (1) 研究の変更  本研究に関する研究実施計画書およびインフォームドコンセント文書の変更または改訂には、研究倫理委員会の事前承認を必要とする。  (2) 研究の中止  以下のいずれかに該当する場合、研究責任者は研究を継続するか検討する。  1） 被験者を募集し、予定症例数に達することが著しく困難と判断した場合。  2） 倫理委員会が実施計画等の変更を指示し、これに応じることが困難と判断した場合。  研究責任者は、研究倫理委員会から研究の中止の勧告又は指示があった場合には、研究を中止しなければならない。 |
| --- |

10. 研究実施期間について記載してください。

| 研究の実施期間は倫理委員会承認後から2024年3月31日までを予定している。 |
| --- |

11. 統計解析方法について記載してください。

| 本研究では、アロマシールとプラセボシールの使用群とで群間比較を実施する。  記述統計は参加者の人口統計的特徴を示すために使用する。平均値と標準偏差は数値データの要約に、頻度と比率はカテゴリデータを要約するために用いる。  アロマシールの利用によるストレスの緩和状況の評価は、DASS-21の各時点のスコアについてベースラインからの変化量を従属変数、DASS-21のベースラインスコアを共変量として、混合効果モデルの共分散分析により行う。  WHO-5の解析は、WHO-5のスコアについてベースラインからの変化量を従属変数、WHO-5のベースラインスコアを共変量として、混合効果モデルの共分散分析により行う。  マスクの着用による息苦しさの評価は、マスク着用による息苦しさについてベースラインからの変化量を従属変数、ベースラインスコアを共変量として、混合効果モデルの共分散分析により行う。  有害事象の発生については群ごとに発生率を算出し、安全性の評価を行う。すべての検定は、両側検定で実施し、有意水準は0.05 未満となるように設定する。 |
| --- |

12. 被験者の人権に対する配慮および個人情報の保護の方法について記載してください。

| 本研究の実施に際し、星薬科大学研究倫理委員会の審査を受けて開始する。本研究の実施にあたってはヘルシンキ宣言（2013年10月フォルタレザ改訂）、人を対象とする生命科学・医学系研究に関する倫理指針（2022年3月10日改訂）ならびに関連法令を遵守する。申請者は星薬科大学研究倫理委員会で承認が得られた研究同意説明書を対象者に渡し、文章及び口頭による十分な説明を行い、対象者の自由意思による研究への参加について同意を文書で得て実施するなどの倫理的配慮を行う。  研究実施に係る情報等を取扱う際は、被験者の個人情報とは無関係の番号を付して管理し、被験者の秘密保護に十分配慮する。また、研究の結果を公表する際は、被験者を特定できる情報を含まないようにする。研究の目的以外に、研究で得られた被験者の試料等を使用しない。 |
| --- |

13. 同意取得方法を記載してください。

| 研究担当者は、審査委員会で承認の得られた同意説明文書を被験者に渡し、文書および口頭による十分な説明を行い、被験者の自由意思による同意を文書で取得する。 |
| --- |

14. 被験者の費用負担について記載してください。

| 本研究は星薬科大学の研究費で賄うため、被験者の費用負担はない。 |
| --- |

15. 記録の保存と研究結果の公表について記載してください。

| 研究責任者は、研究等の実施に関わる重要な文書（申請書類の控え、倫理委員会からの通知文書、各種申請書・報告書の控、被験者識別コードリスト、同意書、その他データの信頼性を保証するのに必要な書類または記録等）を、研究の中止または終了後5年が経過した日までの間保存し、その後は個人情報に注意して廃棄する。本研究の成果を関連学会等において発表することにより公表する場合は、被験者が特定されないよう個人情報の保護に十分配慮する。 |
| --- |

16. 被験者の費用負担について記載してください。

| 本研究は星薬科大学の研究費で賄うため、被験者の費用負担はない。 |
| --- |

17. 研究資金および利益相反について記載してください。

| 本研究に関連し開示すべきCOI関係にある企業などはない。また、本研究を実施するにあたり資金提供は受けていません。研究資金は星薬科大学 教室研究費より支出します。 |
| --- |

18. 研究実施体制について記載してください。

| (1) 研究代表者(プロトコル案（試験計画書）、調査票、同意文書等を作成し、試験の運営および管理において中心的役割を果たす。)  ・湧井 宣行（星薬科大学）  ・市川 ことは（星薬科大学）  (2) 研究データマネージメント監督者（研究代表者と共に本試験の運営に責任を持ち、試験が円滑、かつ公平・中立的に推進される様、監督を行う。）  ・山村 美保（星薬科大学）  (3) 研究協力者  ・外川 知佳子（星薬科大学）  ・松岡 玲虹（星薬科大学）  ・渡邉 舞（星薬科大学）  ・白水 俊介（星薬科大学）  ・吉澤 有紀子（星薬科大学）  (4) 試験統計解析  ・湧井 宣行（星薬科大学）  ・市川 ことは（星薬科大学） |
| --- |

19. 問い合わせ先を記載してください。

| 研究計画の内容に関する問い合わせ先  　　研究実施代表者：  氏名：　湧井　宣行  所属：　星薬科大学　実務教育研究部門  資格：　講師  連絡先  電話：　03-5498-5760  メールアドレス： n-wakui@hoshi.ac.jp |
| --- |
