# Supplementary material for: Evaluating the effectiveness of applying aroma seals to masks in reducing stress caused by wearing masks: A randomized controlled trial
Source: PLoS One. 2023 Nov 16;18(11):e0294357. doi: 10.1371/journal.pone.0294357 (PMC10653515; doi:10.1371/journal.pone.0294357)
Supplement: Attachment — Submitted filename: Response to Reviewers3.docx [file pone.0294357.s009.docx]

**Complementary Therapies in Medicine – Author response form for Manuscript Revisions　# Reviewer3**

**Manuscript Title:** ___Evaluating the effectiveness of applying aroma seals to masks in reducing stress caused by wearing masks: A randomized controlled trial

**PLOS ONE Manuscript Number:** ______PONE-D-23-19767__________________________________

**Instructions:**

Please use this table format to answer the questions posed by the editors and reviewers of your paper. Copy and paste the editor/reviewer’s question in the
“Comments” column and your answer to that question in the corresponding “Response” column. For clarity, use one row per question. Make sure to list the page and line reference where your change can be found. If no change was made, please make sure to note that in your response in addition to your reasoning.

| **Comments**  *Reviewer’s question here.* | **Response**  *Answer to reviewer’s question here. Please respond to each individual comment in a separate row. Responses should be comprehensive and should explain why you do or do not agree with the reviewer’s comment, and what if any action you are taking in response. If a reviewer asks for more information or clarification, you should assume that this new information/clarification should be added to the manuscript (in other words, do not respond in this document but leave the manuscript unchanged).  If text is changed to answer this question, please insert the relevant revised text in your answer, and indicate what text is new with bold or highlighted font.* | **Reference page**  P*age and line number where change can now be found.* |
| --- | --- | --- |
| The detail information on the randomization method including software and allocation concealment and who conducted the randomization process is to be provided. | Thank you for your pointed out. We have added additional details regarding the randomization process as follows:  “**An independent allocation coordinator prepared the aroma and placebo seals, creating an allocation table for each participant, including unique identification codes. Random allocation was conducted by a third party not involved in the study, and allocation results were securely stored in a locked safe. Blinding of intervention providers and data analysts was maintained until the completion of data analysis. Randomization was performed using the R statistical software (version 4.0.2, Foundation for Statistical Computing, Vienna, Austria).**” | **Page5-6**  **Line94-99** |
| For the inclusion criteria, the language criteria (spoken and written) e.g. Japanese is to be added. | Thank you for your feedback. We have made the necessary revisions and included the following statement: "**Eligible participants were those who, to the extent that it did not impede the conduct of the study, had the ability to comprehend the Japanese language, both spoken and written, allowing them to understand the study instructions.**" | **Page5**  **Line82-84** |
| Most questionnaires cited were in English, and I assumed that Japanese version was used in the study. As such, the language version of the questionnaires/survey is to be clearly stated. Any translated version used is to be cited. | Thank you for your feedback. We appreciate the clarification and have added references accordingly for the Japanese versions of the DASS-21 and WHO-5 scales.  We have added the following text regarding the DASS-21: “**In this study, we used the Japanese version of the DASS-21 (Short-form version of the Depression Anxiety Stress Scale) to assess participants' levels of depression, anxiety, and stress [36]. Higher factor scores indicate more-severe depression, anxiety, and stress.**”  Reference for the Japanese version of DASS-21:  **[36] Yuki Murakami, Yukio Imamura, Satoko Mitani, The Short-form Version of the Depression Anxiety Stress Scale (DASS-21) :Reliability and Validity in Female Japanese Undergraduate Students, Journal of Japan Health Medicine Association, 31, 380-389, 2022**  We have added the following text regarding the WHO-5: **“In this study, we used the Japanese translated version of the WHO-5 [37].”**  Reference for the Japanese version of WHO-5:  **[37]** **Psychiatric Research Unit, Mental Health Centre North Zealand. WHO-Five Well-being Index (WHO-5)., [cited 2023 Sep 24] Available from: http://www.who-5.org/** | **Page7**  **Line137-139**  **Page18**  **Line408-410**  **Page7**  **Line150**  **Page18**  **Line411-412** |
| There was no information on sample size calculation. | Thank you for pointed out. We appreciate the reviewer's concern regarding the absence of a sample size calculation in our manuscript and supplementary protocol. While we did not pre-calculate the sample size and relied on a convenience sample, we have now calculated Cohen's d effect sizes for each outcome measure to provide additional insights into the observed effects. We acknowledge this limitation in the manuscript to ensure transparency and have included this information in a limitation part of discussion.  We have added the following content to the limitations section of the study. :“**Additionally, it is important to acknowledge that this study did not pre-calculate the sample size, relying on a convenience sample, which may limit statistical power to detect small effects. This limitation should be considered when interpreting the results and their generalizability.**"  Additionally, We have included a sentence about effect size calculation in the Statistical Analysis section.: “**Additionally, we calculated Cohen's d effect sizes for stress, depression, and other relevant measures to assess the practical significance of the results.**”  We have added the following content to the discussion regarding Cohen's d.: “**Notably, from the value of effect size, the strongest effect was on depression.**” | **Page13**  **Line287-289**  **Page8**  **Line169-170**  **Page14**  **Line259-260** |
| Per protocol analysis to be mentioned. | We would like to express our appreciation for the reviewer's valuable input. In response to the suggestion regarding 'Per protocol analysis to be mentioned,' we have included the following statement in the Statistical Analysis section of the manuscript:  '**Efficacy assessments were performed on participants who successfully completed all trial sessions, while safety assessments were conducted for those who received at least one intervention.**' | **Page8**  **Line170-171** |
| Line 140, what ratio referring to is be clearly stated. | Thank you for your pointed out. The corrected sentence reads as follows: '**Numerical data (age, number of outings per week, and outcome measures) were presented as means and standard deviations, whereas categorical data (sex, stress, and favorite aroma) were presented as frequencies and ratio.**' | **Page7**  **Line153-155** |
| Line 149, the adjusted variables are to be stated apart from baseline. | I have reviewed the areas you pointed out, and it appears that there was an error in the English terminology. What I intended to convey is that the baseline values of each outcome variable are incorporated as covariates. Therefore, I have revised the statement as follows to make it clear that the baseline values of each outcome variable are being used as adjustment variables. I apologize for any confusion caused.  “**DASS-21 and mask-breathlessness scores were analyzed using a mixed model with repeated measures (MMRM) with their respective baseline values as covariates. WHO-5 scores were analyzed using a mixed-effects model analysis of covariance, also incorporating the respective baseline values.**” | **Page7-8**  **Line162-165** |
| Line 158-159, there were discrepancies. It was stated ‘30 and 31 participants in the aroma- and placebo-seal use groups’ respectively but Table 1 and Table 5 it was stated ‘’ Aroma-seal use group (n = 31) Placebo-seal use group (n = 30)’ and ‘Aroma-seal use group (n = 31) Placebo-seal use group (n = 32)’ respectively. | Thank you very much for bringing this discrepancy to our attention, and we sincerely apologize for the error. The accurate participant count is as follows: **the Aroma-seal use group consisted of 31 participants, while the Placebo-seal use group included 30 participants.**  The number of participants **in Table 5 is also the same, with 31 participants in the aroma-seal group and 30 participants in the placebo-seal group.**  We have corrected the relevant parts and tables in the manuscript. Once again, we apologize for any confusion caused. | **Page13**  **Line237 (Table5)**  **Page8**  **Line177-178** |
| Table 1, mean (sd) to be denoted in the table footnote. | Thank you for your pointed out. As per your recommendation, we have added the following to the footnote of Table 1. “**The values in Table 1 for quantitative data represent means ± standard deviations (SD), while qualitative data is presented as frequencies (%).**” | **Page9**  **Line187-188** |
| Table 2, spacing is to be used to space out between each variable and the results. | Thank you for your observation. **We have corrected Table 2 by inserting spaces between each variable and its corresponding outcome. Thank you for your observation.** | **Page10-11**  **Line203 (Table2)** |
| Line 183, the statement ‘All analysis adjusted for baseline value, Time, Time and baseline * Time.’’ is to be mentioned in the statistical analyses section. | Thank you for your pointed out. Due to the unclear wording, we have removed the relevant section and rephrased it as follows: “**DASS-21 and mask-breathlessness scores were analyzed using a mixed model with repeated measures (MMRM) with each outcome variable baseline values as covariates. WHO-5 scores were analyzed using a mixed-effects model analysis of covariance, also incorporating each outcome variable baseline values.**” Furthermore, we have removed the sentence below the tables. | **Page7-8**  **Line162-165** |
| Table 4 title, the week assessment to be added. | Thank you for your pointed out. We have corrected Table 4 title as follows.  “**Table 4. Comparison of WHO-5 score changes from baseline at the end of the two-week intervention in the aroma- and placebo-seal groups.**” | **Page12**  **Line226-227** |
| Table 2, 3 and 4, n to be stated. | Thank you for pointing that out.  We have added **'n' to Tables 2, 3, and 4.** | **Page10 Table2**  **Page11 Table3**  **Page12 Table4** |
| Data for baseline/first week/second week of intervention of DASS-21, mental well-being etc is to be provided prior displaying ANCOVA analysis output. | Thank you for pointing that out. We have created Supplementary Tables “**S1 Table**” and “**S2 Table**” for DASS-21 and WHO-5, respectively.  At the beginning of the DASS-21 results section, we noted,“**DASS-21, which assesses depression, anxiety, and stress, was utilized in our study. In its evaluations at baseline, 1 week, and 2 week, the scores for the Aroma-Seal Use Group appeared to show a decreasing trend (S1 Table).**”  At the beginning of the WHO-5 results section, we noted, “**The average score for each item of the WHO-5 was observed to be higher in the intervention group compared to the placebo group (S2 Table).**“ | **Page10**  **Line195-196**  **Page11**  **Line220-221** |
| Alignment columns for some tables requires adjustment to ensure the letters are not separated apart. | Thank you for your feedback. **We have** **made adjustments to prevent the letters from separating in Tables 1, 4, and 5**. | **Page8 Table1**  **Page12 Table4**  **Page13 Table5** |
